# Proinflammatory profile of in vitro monocytes in the ageing is affected by lymphocytes presence

**DOI:** 10.1186/1742-4933-10-22

**Published:** 2013-06-08

**Authors:** Karen Henriette Pinke, Bruno Calzavara, Patricia Freitas Faria, Magda Paula Pereira do Nascimento, James Venturini, Vanessa Soares Lara

**Affiliations:** 1 Department of Stomatology, Bauru School of Dentistry, University of São Paulo, Al. Dr. Octávio Pinheiro Brisola, 9-75, 17012-901, Bauru, SP, Brazil; 2 Laboratory of Experimental Immunology, Department of Biological Sciences, Faculty of Science, São Paulo State University, Av Eng Luiz Edmundo C, 14-01, 17033-360, Bauru, SP, Brazil

**Keywords:** Immunosenescence, Monocytes, PBMC, Cytokines, *Inflamm-aging*

## Abstract

**Background:**

Aging is associated with complex and constant remodeling of the immune function, resulting in an increasing susceptibility to infection and others diseases. The infections caused by Gram-negative microorganisms, present in nursing homes and hospitals, constitute one of the most common infections in the elderly, and are mainly combated by innate immune cells. Although the functions of innate immunity seem more preserved during aging than of adaptive immune mechanisms, two systems operate in an integrated way in the body, so that injury in one part of the immune system inevitably affects the other as they are part of a defensive network. The aim of this study was to investigate the *in vitro* production of proinflammatory (TNF-α, IL-6, IL-1β, CXCL-8 and MCP-1) and anti-inflammatory (TGF-β and IL-10) cytokines by monocytes, stimulated or not (basal) with lipopolysaccharide, from healthy young and elderly subjects. By means of PBMCs, we also studied if cytokine profile is altered in these different patient groups, in the presence of lymphocytes, under the same experimental conditions.

**Results:**

The monocytes from elderly presented higher basal production of TNF-α, MCP-1 and lower of TGF-β than young monocytes. PBMC showed similar cytokines production, irrespective age or stimulation presence. In the presence of lymphocytes, the spontaneous production of IL-10 was higher and of TGF-β was lower than monocytes, regardless of age. After LPS-stimulation, the presence of lymphocytes resulted in increased IL-6, IL-1β, MCP-1 and IL-10 and decreased CXCL-8 and TGF-β in comparison to pure culture of monocytes from young patients. With age, the same differences were observed, except for CXCL-8 and TGF-β which production was the same between monocytes and PBMC stimulated with LPS.

**Conclusion:**

These findings reinforce the systemic state of *inflamm-aging* frequently reported in elderly and considered a factor of susceptibility to numerous diseases. Still, the cytokine production from just monocytes of the elderly showed alterations, while in the lymphocyte presence not, suggesting an immunomodulator role of lymphocytes on monocytes. In addition, the differences between the production patterns by LPS-stimulated PBMC between young and elderly volunteers can be related with an imbalance in response against Gram-negative bacteria in throughout life.

## Background

In recent decades, the number of elderly has increased considerably in comparison with young people and there is a trend towards continuing increase in the twenty-first century [1]. With these changes in life expectancy of the population, there has been increasing interest in studies related to health and quality of life during aging. Advancing age affects various cellular and biological functions, including immune cells [2-9], mostly related to changes in the equilibrium between cell survival, proliferation and death. In vitro experiments have shown that absence of the enzyme telomerase leading to critical shortening of the protective ends of chromosomes plays a central role in human cell senescence [10,11]. These changes in immunosenescence are related to various impairments such as susceptibility to inflammatory, infectious and autoimmune diseases, as well as increasing rates of occurrence of malignancies [1,12-14]. Although these diseases are usually easily resolved clinically, elderly humans suffer from complications caused by the individual's altered response [1]. Much of this deterioration of immunity in elderly is related to predisposition to infectious diseases, such as caused by Gram-negative microorganisms that are frequent in nursing homes and hospital settings [15,16] and mainly combated by innate immune cells. Although the functions of innate immunity appear to be more preserved during aging than the mechanisms developed by the adaptive immune system [8,17], recent evidence has shown that most innate immune functions are at least partly affected during aging [5-7,18-20]. Thus, changes related to immunosenescence could affect the defense mechanism as a whole; however, this complex process is not yet completely understood.

Mononuclear phagocytes cells compromise macrophages and their monocyte precursors and linage-committed bone marrow precursors. Both macrophages and peripheralblood monocytes exhibit morphological heterogeneity and functional plasticity [21]. Lipopolysaccharide (LPS), an endotoxin present in the cell wall of Gram-negative bacteria and widely used for immunological assay, is recognized by monocyte/macrophage CD14 receptor and toll-like receptor (TLR) 4-MD2 [22]. These molecular interactions result in the release of different active molecules in various inflammatory mechanisms including interleukin-1 (IL-1), prostaglandin E2 (PGE2) and tumor necrosis factor alpha (TNF-α) [22,23], which may be further amplified by lymphocytes, the main source of important cytokines such as interferon-gamma (IFN-γ) [24-26]. Peripheral blood mononuclear cells (PBMC) are composed by monocytes and lymphocytes and represent an important line of defense against infection [27-30]. Moreover, human PBMC culture is a classical and well-known test for evaluation of the immunological status and for mimicking changes that occur in infectious lesions and tissue remodeling and repair after inflammation [21].

During immunosenescence, the role of T lymphocytes is modified and related mainly to changes in intracellular signaling pathways [31], with decreased generation of lymphoid precursors [1,32] and their ability to proliferate [14]. With advancing age, changes in monocytes are mainly related to decline in the production of cytokines such as IL-1, and decrease in tumoricidal activity and superoxide production [33]. Whereas, monocytes from aged individuals are associated with increased levels of circulating interleukin-6 (IL-6) and TNF-α [34], characterizing the state called *inflamm-aging*, i.e., chronic systemic inflammation in the elderly [14]. This persistent pro-inflammatory profile results from high antigenic exposure throughout life to various microorganisms, including bacteria, which can change macromolecules such as deoxyribonucleic acid (DNA) or proteins by oxidation, acylation or glycosylation. Theses altered molecules can stimulate the innate immune response, particularly macrophages via TLRs [35].

The better understanding of the changes in immunomodulatory interplay between lymphocytes and monocytes during aging and in the cooperation between the cells in this defense process could elucidate the immune mechanisms of the elderly associated with increased susceptibility to infectious diseases, especially those caused by Gram-negative bacteria [1,14-16,36]. Therefore, this study investigated the in vitro production of cytokines TNF-α, IL-6, IL-1β, CXCL-8, MCP-1, TGF-β and IL-10 by PBMC and monocytes from elderly and young volunteers after LPS stimulation.

## Results

**Figure 1:**
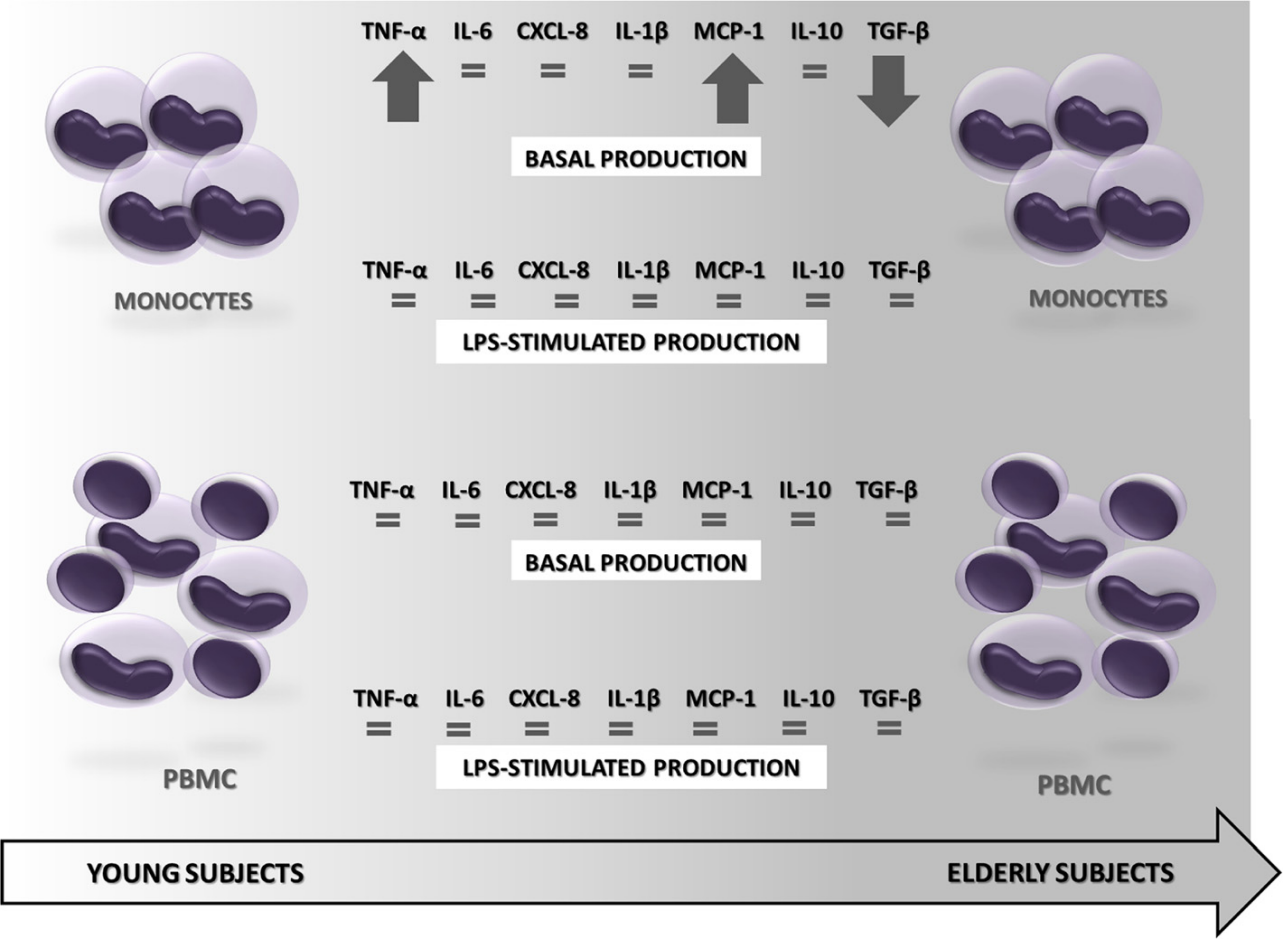
**Main differences between the cytokines production by monocytes and PBMC from elderly subjects in relation to young.** With increasing age, monocytes showed higher basal production of TNF-α, MCP-1 and lower of TGF-β than monocytes from young.

**Figure 2:**
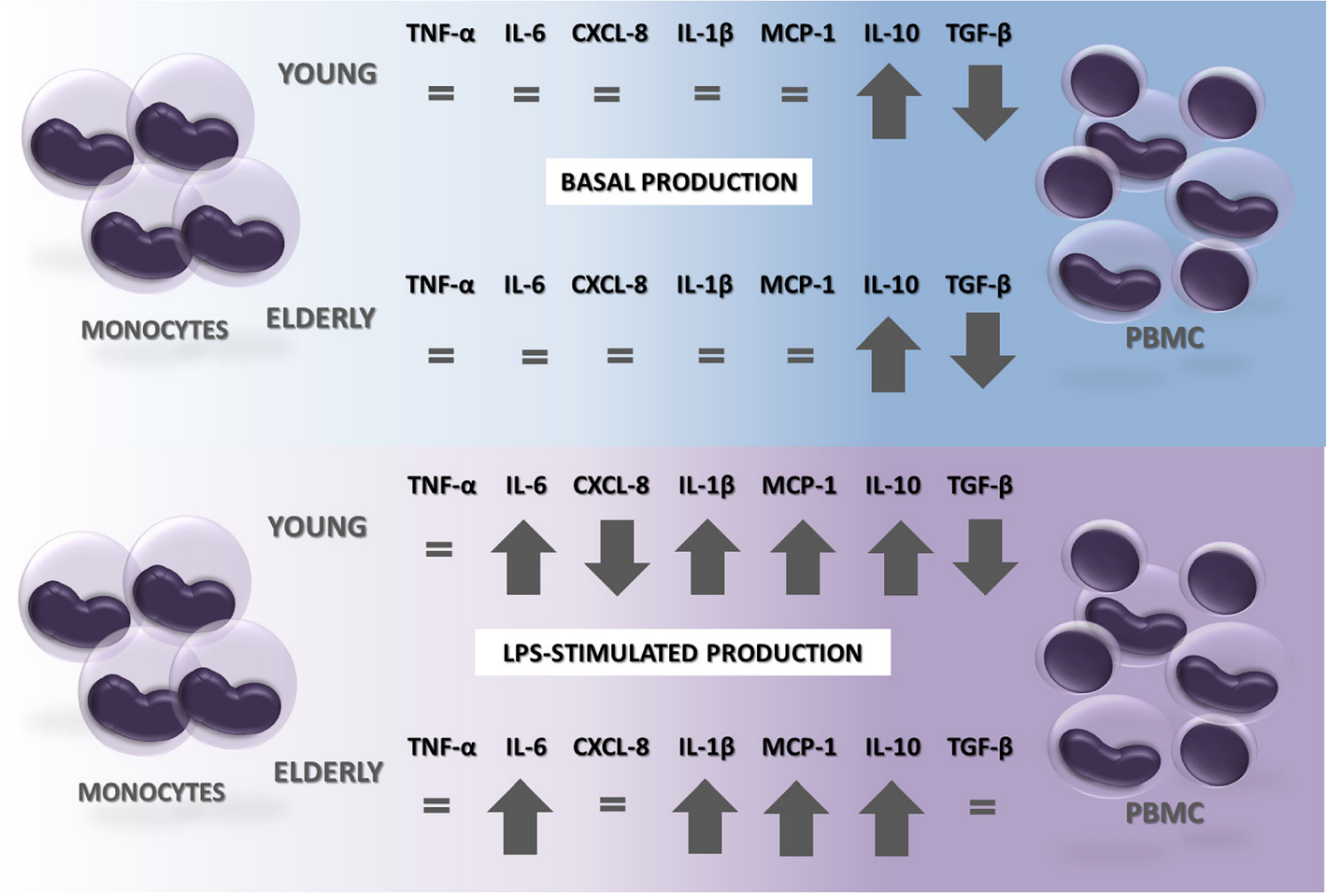
**Influence of lymphocytes presence on cytokines production.** Note that with the presence of lymphocytes the spontaneous production of IL-10 was higher and of TGF-β was lower than that of monocytes, regardless of age. After stimulation with LPS, the presence of lymphocytes resulted in increased IL-6, IL-1β, MCP-1 and IL-10 and decreased CXCL-8 and TGF-β in comparison to pure culture of monocytes from of young patients. With age, the same differences were observed, except for CXCL-8 and TGF-β which production was the same between monocytes and PBMC stimulated with LPS.

**Figure 3:**
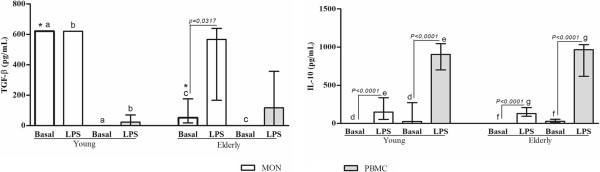
**Measurements of the anti**-**inflammatory cytokines production by blood monocytes (MON) or PBMC from healthy elderly and young subjects.** Peripheral blood was obtained from volunteers, and the cells were purified as described in the Material and Methods section. Monocytes and PBMC were challenged with LPS (100 ng/mL), or not (Basal), for 18 and 24 hours, respectively, and cytokine production was determined by ELISA. Columns represent the median and error bar the interquartile range. The results were evaluated by Mann–Whitney Rank Sum Test. In the same subject group, statistical differences are represented by continuous lines (cells stimulated or not) and letters for different cell types. Asterisks indicate statistical difference between groups of subjects (*p* values - ^*^0,0016; ^a and b^ 0,0039; ^c^ 0,0159; ^d^ 0,0010, ^e, f and g^ <0.0001).

Monocytes from elderly subjects spontaneously produced more TNF-α, MCP-1 and less TGF-β than young. The values of cytokine levels detected from monocytes or PBMC obtained from young or elderly persons were demonstrated as median, minimum, maximum and 1^th^, 3^th^ quartiles (Tables 1 and 2). The main findings are schematized in Figures 1 and 2. The basal production of TNF-α and MCP-1 by monocytes from elderly group was higher than those derived from young individuals. However, the basal TNF-α production by PBMC showed a fall in elderly, becoming below than young, although without statistical difference (Tables 1 and 2). On the other hand, the TGF-β production without stimulus by monocytes from elderly was lower than young, while this production was not detected by PBMC from all subjects (Figure 3).

**Table 1:** Cytokines production by monocytes or PBMC from young subjects

**Cytokines**	**Cellular type**	**Production type**	**Median**	**Minimum**	**Maximum**	**1th quartile**	**3th quartile**
**TNF-α**	**Monocyte**	**Basal**	0.0	0.0	24.4	0.0	0.0
		**LPS**	246.6	34.5	1486.5	182.8	97.6
	**PBMC**	**Basal**	2.5	0.0	19.0	0.0	10.4
		**LPS**	341.6	70.8	1165.8	270.3	728.7
**IL**-**6**	**Monocyte**	**Basal**	2.6	0.0	68.1	0.0	8.9
		**LPS**	175.8	50.5	282.0	138.3	186.1
	**PBMC**	**Basal**	0.0	0.0	123.2	0.0	6.4
		**LPS**	139.6	0.0	266.6	116.7	218.2
**IL**-**1β**	**Monocyte**	**Basal**	0.0	0.0	0.1	0.0	0.0
		**LPS**	69.6	15.3	173.7	37.5	173.7
	**PBMC**	**Basal**	0.0	0.0	1.0	0.0	0.0
		**LPS**	17.9	0.0	113.8	0.0	31.5
**CXCL**-**8**	**Monocyte**	**Basal**	173.1	48.7	288.8	130	240.1
		**LPS**	279.5	177.3	297.7	252.3	290.9
	**PBMC**	**Basal**	144.8	64.0	215.1	118.6	185.4
		**LPS**	229.8	218.4	305.4	219.8	303.0
**MCP**-**1**	**Monocyte**	**Basal**	6.4	0.0	190.5	0.0	39.8
		**LPS**	48.2	0.0	241.2	0.0	148.4
	**PBMC**	**Basal**	25.9	5.9	412.4	12.2	97.5
		**LPS**	85.0	0.0	415.3	29.1	172.7
**TGF**-**β**	**Monocyte**	**Basal**	621.2	621.1	621.4	621.1	621.3
		**LPS**	621.2	621.0	621.6	621.1	621.2
	**PBMC**	**Basal**	51.7	8.9	220.5	25.8	130.9
		**LPS**	567.2	110.2	690.5	223.1	587.9
**IL**-**10**	**Monocyte**	**Basal**	0.0	0.0	5.9	0.0	0.0
		**LPS**	149.3	33.8	654.8	55.4	323.8
	**PBMC**	**Basal**	0.0	0.0	11.8	0.0	0.0
		**LPS**	129.1	47.0	274.1	102.7	195.6

Values showed by median, minimum, maximum, 1^th^ and 3^th^ quartiles obtained by descriptive statistical analysis (Statsoft Software Inc. Tulsa, Ok, USA).

**Table 2:** Cytokines production by monocytes or PBMC from elderly subjects

**Cytokines**	**Cellular type**	**Production type**	**Median**	**Minimum**	**Maximum**	**1th quartile**	**3th quartile**
**TNF**-**α**	**Monocyte**	**Basal**	0.0	0.0	253.1	0.0	98.4
		**LPS**	489.8	141.6	935.2	378.8	572.2
	**PBMC**	**Basal**	0.0	0.0	98.4	0.0	0.0
		**LPS**	556.1	336.3	1425.1	433.0	694.7
**IL**-**6**	**Monocyte**	**Basal**	0.0	0.0	0.1	0.0	0.1
		**LPS**	363.2	319.8	391.8	329.4	389.6
	**PBMC**	**Basal**	0.0	0.0	0.1	0.0	0.1
		**LPS**	321.1	246.4	489.4	283.2	405.8
**IL**-**1β**	**Monocyte**	**Basal**	0.3	0.0	6.8	0.0	3.7
		**LPS**	332.5	276.1	362.3	293.3	358.3
	**PBMC**	**Basal**	0.0	0.0	12.3	0.0	6.1
		**LPS**	323.0	280.0	336.7	299.9	331.4
**CXCL**-**8**	**Monocyte**	**Basal**	59.5	9.3	213.2	11.8	158.9
		**LPS**	202.8	191.1	219.6	193.3	214.8
	**PBMC**	**Basal**	160.1	16.7	197.5	82.3	184.9
		**LPS**	206.5	200.6	224.5	200.7	218.4
**MCP**-**1**	**Monocyte**	**Basal**	4.6	0.0	125.1	0.0	125.1
		**LPS**	999.6	988.0	1039.2	988.0	1039.2
	**PBMC**	**Basal**	314.8	85.2	362.3	85.2	362.3
		**LPS**	994.4	948.2	1013.4	948.2	1013.4
**TGF**-**β**	**Monocyte**	**Basal**	0.0	0.0	0.1	0.0	0.1
		**LPS**	23.3	0.0	78.6	0.0	62.6
	**PBMC**	**Basal**	0.0	0.0	0.1	0.0	0.1
		**LPS**	117.0	0.0	397.8	0.0	315.9
**IL**-**10**	**Monocyte**	**Basal**	24.6	0.0	358.6	0.0	271.2
		**LPS**	903.4	447.2	3115.4	701.3	1044.9
	**PBMC**	**Basal**	28.1	0.0	508.2	12.2	52.4
		**LPS**	966.8	490.3	1139.2	649.8	1031.2

Values showed by median, minimum, maximum, 1^th^ and 3^th^ quartiles obtained by descriptive statistical analysis (Statsoft Software Inc. Tulsa, Ok, USA).

**Figure 4:**
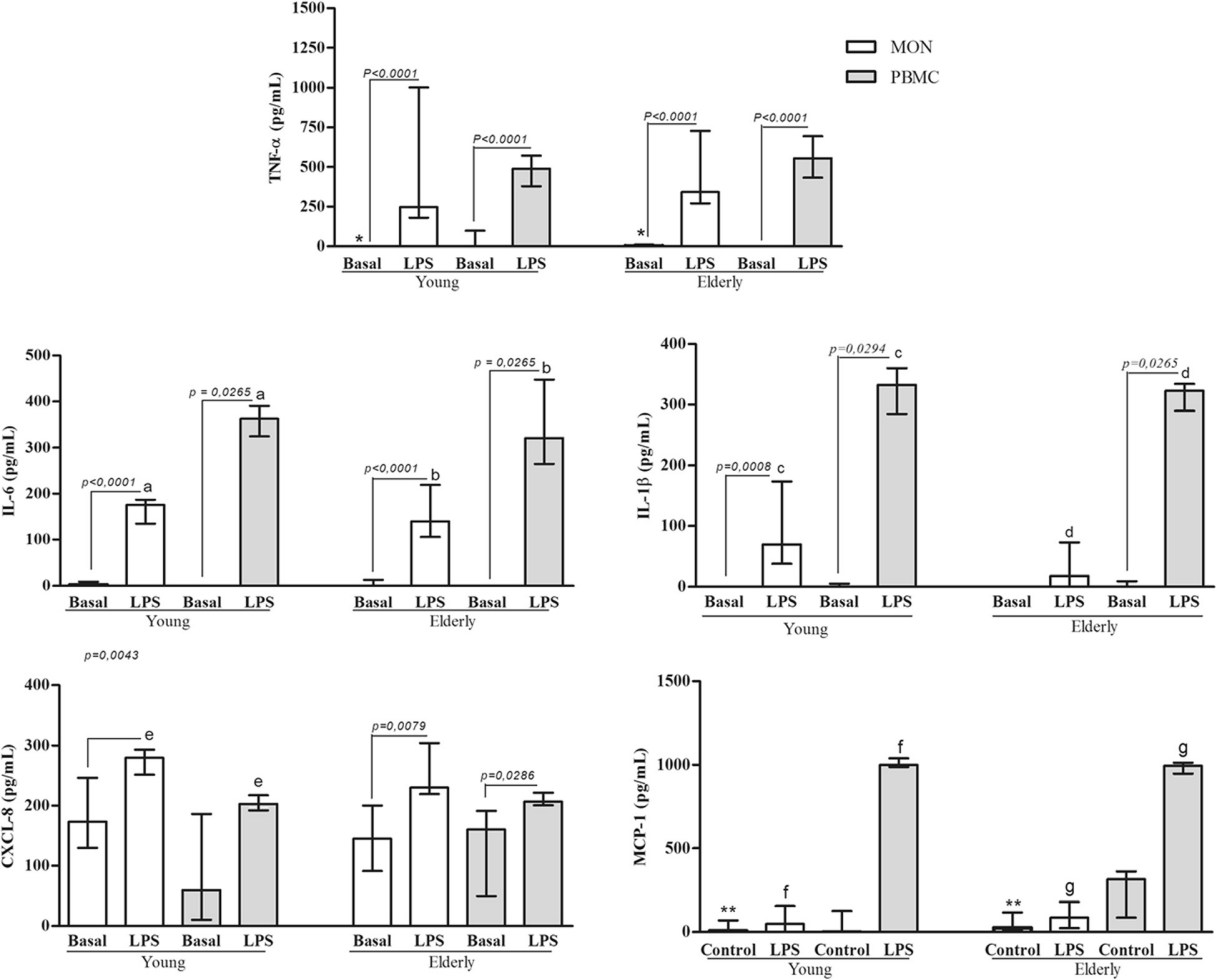
**Measurements of the proinflammatory cytokines production by blood monocytes (MON) or PBMC from healthy elderly and young subjects.** Peripheral blood was obtained from volunteers, and the cells were purified as described in the Material and Methods section. Monocytes and PBMC were challenged with LPS (100 ng/mL), or not (Basal), for 18 and 24 hours, respectively, and cytokine production was determined by ELISA. Columns represent the median and error bar the interquartile range. The results were evaluated by Mann–Whitney Rank Sum Test. In the same subject group, statistical differences are represented by continuous lines (cells stimulated or not) and letters for different cell types. Asterisks indicate statistical difference between groups of subjects (*p* values - *0,0487; ^a^ 0,0029; ^b^ 0,0068; ^c^ 0,0061; ^d^ 0,0195; ^e^ 0,0180; ^**^ 0,0399; ^f^ 0,0089; ^g^ 0,0098).

Although the basal production has been different in some conditions between young and elderly subjects, LPS-stimulated monocytes and PBMC from both age groups produced similar cytokines levels (Figures 1, 3 and 4).

In PBMC, LPS increased more the cytokines production than in monocytes. In the most experimental conditions, the LPS caused increase of the cytokine levels in relation to basal production, except MCP-1 and TGF-β (Figures 3 and 4).

On the comparative analysis within age groups, in the presence of lymphocytes, the spontaneous production of IL-10 was higher and of TGF-β was lower than that of monocytes, regardless of age. However, LPS-stimulated PBMC from young or elderly produced more IL-1β, IL-6, MCP-1 and IL-10 than its respective monocytes (Figures 2, 3 and 4). Besides, the presence of lymphocytes under the LPS-stimulation resulted in lower production of CXCL-8 and TGF-β in comparison to pure culture of monocytes in young but not in elderly (Figures 2, 3 and 4).

## Discussion

According to the vitro results of this study addressing cytokines, the monocytes suffer changes throughout life. The spontaneous production of the TNF-α and MCP-1 by monocytes were increased in aged volunteers compared with those of young individuals. However, the TGF-β basal production was lower with senescence. These immune differences between young and aged people may represent some aspects of the complex and constant remodeling of the immune system with advancing age, and contribute with a proinflammatory profile. Among the variations already found and consistent with our results is a high TNF-α concentration detected in the plasma from the elderly [2,37,38], which may be related to the development of acute and chronic inflammatory conditions, carcinogenesis, and autoimmune diseases [1,12-14]. Irrespective of age, the TNF-α dysregulation could be associated to several diseases, such as diabetes type II, rheumatoid arthritis and atherosclerosis, among others [39]. Although this change in the pro-inflammatory profile appears to represent an evolutionary programming to infections resist [40], we not found a relationship between high levels of TNF-α and increased protection against the Gram-negative microorganisms in elderly. MCP-1 is a chemokine that regulates the monocyte and memory T cells migration to sites of antigen-induced inflammation [27,41,42] and is produced mainly in response to inflammatory stimulus [43-45]. Increased levels of MCP-1 in elderly have been correlated with the preservation of the memory T cell population and progression of atherosclerosis [46,47]. Our findings about the increase on basal production of the MCP-1 in elderly could represent a systemic alteration in the traffic of the monocytes and the memory T cells with aging.

Unlike our findings, other studies showed higher levels of TGF-β from macrophages with aging [48,49]. This cytokine have a complex and pleiotropic activity, presenting anti-inflammatory action on various cell types, like mast cells, T cells [50-53] and proinflammatory function on monocytes, attracting to the local aggression and inducing the release of IL-1 and IL-6 by these cells [50,54].

Taken together, these results about spontaneous production of cytokines, obtained from elderly subjects corroborate the state of *inflamm-aging* reported in others works [55-57], which appears to predispose elderly persons to diseases such as Alzheimer's, angina and osteoporosis [58-60]. Besides, it could alter the defense mechanisms against microorganisms [57,61] and reduce the ability to provide the immediate response to pathogens, as well as integrate and influence the acquired immune response [61].

Regarding PBMC, our analyzes showed no significant difference between the basal production of cytokines by elderly in relation to young. Already in stimulated-conditions, PBMC produced higher levels of IL-1β, IL-6, MCP-1 and IL-10 than matched monocytes, irrespective of age. However, CXCL-8 and TGF-β production from stimulated PBMC was lower than matched monocytes just in young group. Some trends involving these production patterns in elderly also can be highlighted. The lymphocytes seem to increase the TNF-α and MCP-1 spontaneous production in the elderly, but not in young. Besides, PBMC from young tend to produce more MCP-1 and less CXCL-8, whereas in advance of age, these cells seem to produce more TNF-α and TGF-β. Thereby, our dates indicate interplay between the two cell types that may have altered the production of cytokines from monocytes, suggesting an immunomodulador role of lymphocytes on monocytes. Also, the modified production pattern after LPS stimulus in the presence of the lymphocytes can indicate a different modulation in the elderly than in the young, under infectious conditions caused by Gram-negatives.

These changes may be related to changes already characterized in lymphocytes with aging. Among them is the variation in lymphocyte population in PBMC. Whereas PBMC from young subjects presents 40,6% of the naïve, 36,6% of the memory and 16,8% of the effector/cytotoxic T cells, in elderly, the lymphocyte population more numerous is of the memory CD8+ T cells (54%) with great reduction on naïve T cells population (3,6%), [62,63]. Thus, despite the phenomena of immunosenescence being more related to the cells of the specific immunity system [8,17], the findings of the present study may represent an important change in innate immunity cells from the elderly, modulated by cells of the specific immunity system, strengthening the hypothesis that the interaction between cells is an important step affected during remodeling of the immune system in advancing age. Studies on immunosenescence have shown controversies, including a decreased production of interleukin-12 (IL-12) by the monocytes of elderly persons in comparison with those of young persons, and similar production of IL-10 by monocytes from young or elderly persons [2,33,34,64-66]. The results of the present study also revealed similar levels of IL-10 produced by monocytes from young and elderly persons, unlike TNF-α. Higher IL-10 production by PBMC compared with isolated monocytes was expected, since lymphocytes are major producers of IL-10. Thus, we tend to believe that the most important alterations observed in the present study could be attributed to monocytes, particularly in the presence of interaction with lymphocytes. Other alterations in T cell populations in the elderly [67-69] cannot be ruled out. Further studies will be needed for better clarification of the molecular mechanisms involved in interaction process between immune cells and pathogens, in the elderly.

## Conclusions

In summary, it was found that in vitro monocytes from aged volunteers spontaneously produced more TNF-α, MCP-1 and less TGF-β in comparison with those from young persons, corroborating the state of *inflamm-aging* capable of predisposing persons to numerous diseases. However, in presence of lymphocyte the cytokines levels were equal between age groups, even after LPS challenge, suggesting an immunomodulador role of lymphocytes on monocytes. Still, after LPS challenge, the pattern of cytokines production in presence of lymphocyte was different between age groups, which may suggest an imbalance in the response against Gram-negative bacteria. Thus, our data may indicate that cooperation between lymphocytes and monocytes from peripheral blood can decrease a basal state of inflammation present the elderly. However in infectious conditions, this modulation can be altered and lead to a lower monocyte recruitment, proliferation and differentiation of other cell types in elderly.

## Methods

### Sample population

Elderly and young adults were recruited from the Bauru School of Dentistry, University of São Paulo (USP), and enrolled in the study. Immunocompromised subjects were excluded, including individuals with human immunodeficiency virus infection (HIV), diabetes mellitus requiring medication, and those taking immunomodulating medications. Subjects who were pregnant, smokers and those under treatment with antipsychotic drugs were also excluded. This study was approved by the Ethics committee at the USP (Protocol No.100/2008, 051/2008 and 172/2009). Informed consent was obtained from all volunteers in compliance with Resolution 196/96 of the National Council of Health. The inclusion criteria comprised healthy subjects in ranging from 20 to 50 years old (young) and from 60 to 85 years old (elderly) [70].

### PBMC isolated

PBMC were obtained by centrifugation of heparinized venous blood over Histopaque 1083 gradients (Sigma-Aldrich, St. Louis, MO, USA) (400 × g for 28 minutes). The cell suspensions recovered at the interface were washed and resuspended in complete medium (RPMI 1640 supplemented with 10% heat-inactivated fetal bovine serum, 100 U/mL penicillin and 100 μg/mL streptomycin). Cell viability, as determined by 0.2% trypan blue dye exclusion, was > 95% in all experiments. PBMC were counted with the Türk dye staining, and suspended in complete medium at a concentration of 1×10^6^ PBMC/mL.

### Monocytes isolated

To obtain the monocyte (MON) suspension, PBMC were isolated as previously described. The monocytes were counted using neutral red (0.02%), and were suspended in complete medium at a concentration of 1×10^6^ monocytes/mL. After this, were distributed (1.0 mL/well) in 24-well plates with a flat-bottomed coverslip in each well to allow adherence of monocytes and immunofluorescence testing on the glass coverslips. After incubation for 2 h at 37°C in a humidified atmosphere of 5% CO_2_, non-adherent cells were removed by aspiration and each well was rinsed twice and resuspended in complete medium. Cell viability was assessed by morphological analysis of the cells on coverslips with marking using the anti-CD14-FITC and labeling of the cell nucleus via DAPi. Over 98% of viable cells were adherent monocytes that had intensely expressed the CD14 receptor, and were morphologically viable.

### Cell challenge with lipopolysaccharide (LPS)

Monocytes and PBMC were incubated with (LPS) or without (Basal) LPS of Escherichia coli O55B5 (Sigma-Aldrich, St. Louis, MO, USA) (100 ng/mL - Mon; 1 ng/mL-PBMC) for 18 and 24 h, respectively, at 37°C in 5%CO_2_. Culture supernatants were harvested and stored at -80°C until assayed.

### Quantification of cytokines

Cytokine concentrations were determined in cell-free supernatants obtained after 24 h PBMC cultures and 18h monocyte cultures, with or without LPS. The method utilized was enzyme-linked immunosorbent assay (ELISA), using BD OptEIA^™^ kits (BD Biosciences) for TNF-α, IL-6, IL-1β, CXCL8, MCP-1, IL-10 and TGF-β. The evaluations were performed according to the manufacturer’s instructions.

### Statistical methods

All dates showed *p* values of less than 0.05 for the Normality Test (Shapiro-Wilk) and were considered non-parametric. For statistical analysis, was utilized the Mann–Whitney Rank Sum Test with *p* values <0.05 considered significant. The software Statistica 11.0 (Statsoft Software Inc. Tulsa, Ok, USA) was used.

## Competing interests

The authors have no financial conflict of interests.

## Authors’ contributions

KHP carried out the immunoassays, assay development, data analyses, statistical analyses, and drafted the manuscript. BC participated of the immunoassays and assay development. PFF participated in the design of the study and helped with editing of the manuscript. MPPN helped to immunoassays, assay development and to draft the manuscript. JV helped in the assay development and helped to draft the manuscript. VSL conceived of the study, participated in your design and coordination and helped to draft the manuscript. All authors read and approved the final manuscript.
